# A large sublingual epidermoid cyst with parapharyngeal space extension: A case report

**DOI:** 10.1016/j.ijscr.2020.06.014

**Published:** 2020-06-11

**Authors:** Hanpon Klibngern, Chanisa Pornchaisakuldee

**Affiliations:** a Division of Head and Neck Surgery and Oncology and Hyperbaric Oxygen Therapy, Department of Otolaryngology, Chiang Mai University, Chiang Mai, Thailand; b Division of Pediatric Otolaryngology, Department of Otolaryngology, Chiang Mai University, Chiang Mai, Thailand

**Keywords:** cm, centimeter, MRI, magnetic resonance imaging, Epidermoid cyst, Transoral, Sublingual, Case report

## Abstract

**Introduction:**

Epidermoid and dermoid cysts in the floor of the mouth are uncommon. A large cyst can lead to functional morbidities including airway, swallowing and speech problems. The treatment of choice is surgical excision. To obtain a good result, the decision for optimal surgical technique is valuable.

**Case presentation:**

A young female patient presented with a slowly growing mass at the submandibular area and swelling in the floor of mouth. The MRI showed large sublingual cyst extended posteriorly to the parapharyngeal space. She underwent surgical excision of the cyst size 6.5 × 3.2 × 2.5 cm via an intraoral approach. The pathological examination revealed a benign stratified squamous epithelium-lined cyst with no evidence of adnexal structure consistent with an epidermoid cyst. The patient remained disease-free after two years of follow-up.

**Discussion:**

Generally, large sublingual cysts over 6 cm located or transgress below the mylohyoid muscle are removed by an extraoral approach. Our team reported the first case of a large lateral sublingual epidermoid cyst with parapharyngeal extension removed by an intraoral approach. Preoperative imaging is important for surgical approach selection.

**Conclusion:**

Successful management of sublingual epidermoid cyst extended to the parapharynx is feasible by an intraoral approach with excellent functional and cosmetic outcomes.

## Highlights

• Large sublingual epidermoid cyst with parapharyngeal space extension.
• Investigation by MRI and FNA provide information for surgical approach.
• Surgical excision is the treatment of choice.
• Sublingual gland removal facilitates intraoral excision.
• An intraoral approach provided excellent cosmetic and functional outcomes.

## 1 Introduction

Epidermoid cyst in the floor of the mouth is an uncommon developmental lesion. The pathogenesis is the entrapment of the ectodermal tissue along embryonic fusion lines. The clinical presentation is a progressive swelling in the floor of the mouth and neck. If large enough, the cyst can potentially compromise the airway, speech, and swallowing. The main treatment is surgical excision by either intraoral or extraoral approach depend on the size and location of the cyst.

We present the case of a large sublingual epidermoid cyst with parapharyngeal space extension excised by an intraoral approach. The patient was managed at our academic institution. The present case is reported in accordance with the SCARE criteria [1].

## 2 Case presentation

**Fig. 1:**
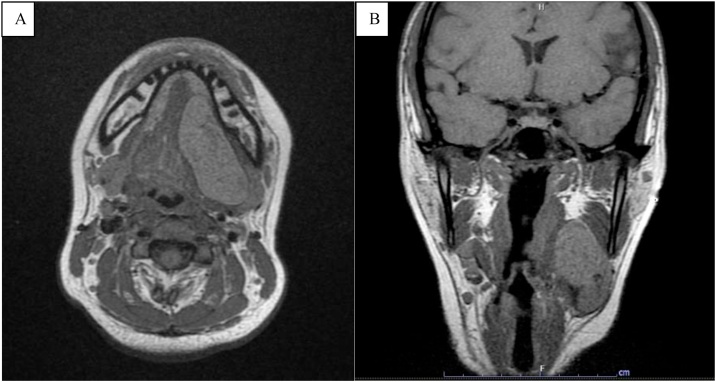
MRI of axial view (A) and coronal view (B) demonstrates the left sublingual epidemoid cyst extended posteriorly to the left parapharyngeal space.

**Fig. 2:**
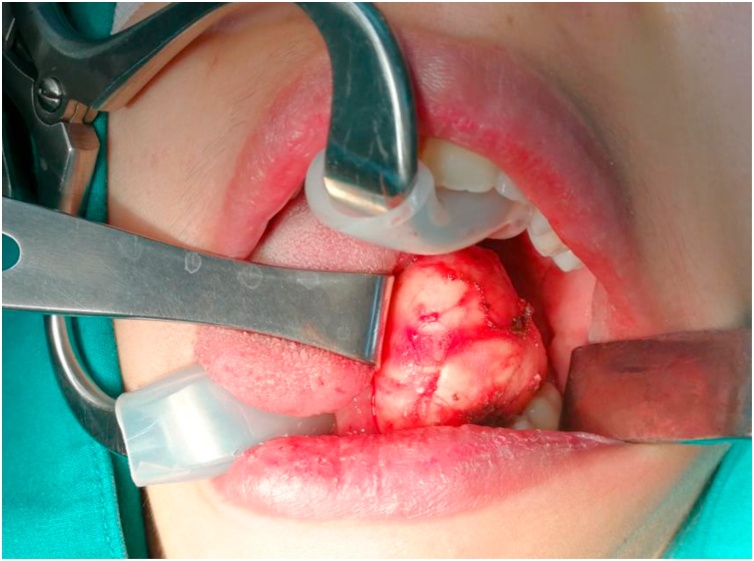
Intraoral excision sublingual epidermoid cyst.

**Fig. 3:**
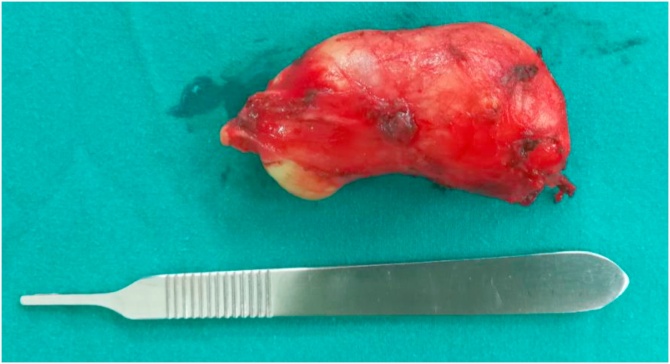
Surgical specimen.

**Fig. 4:**
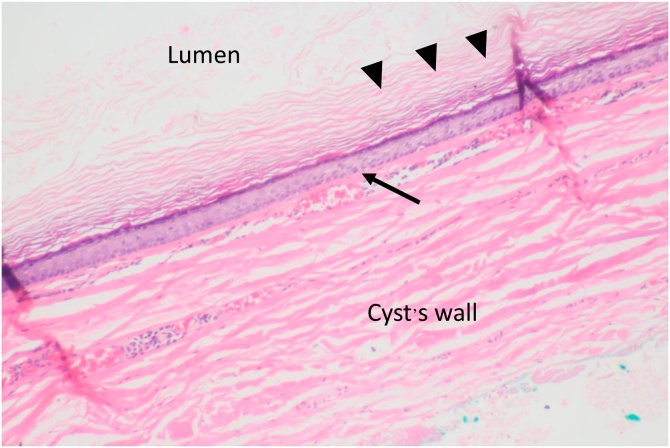
Histopathology of the cyst revealed benign stratified squamous epithelium-lined cyst with no evidence of adnexal structure consistent with the epidermoid cyst, arrowhead: Keratin material, arrow: Squamous epithelial cell (H&E;100x).

**Fig. 5:**
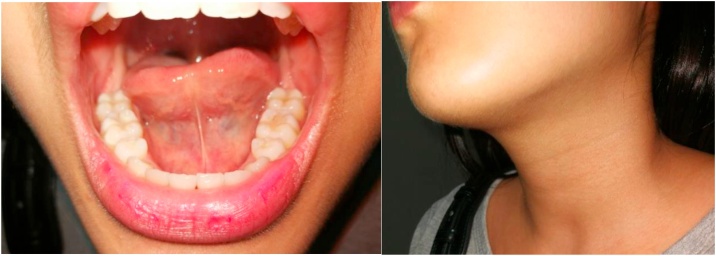
Postoperative photo at one month.

A 26-year-old-female presented with a painless, slowly growing mass over 6 months at the left submandibular area. She reported no increased swelling of the mass or pain during eating. Her speech and swallowing were normal. She had taken two courses of antibiotics from other hospitals, but her symptoms persisted. There was no previous history of surgery or trauma to the oral cavity or neck. Clinical examination revealed a well-defined, firm, non-tender mass size 4 × 4 cm in the left submandibular area. Bimanual palpation of the left submandibular gland showed no stone or pus from the Wharton’s duct. Oral cavity examination revealed swelling in the left floor of mouth with normal tongue movement. We scheduled her for MRI imaging and fine-needle aspiration (FNA) for further investigation. The MRI showed a well-defined hypointense T1 and hyperintense T2-weighted image cystic lesion at the left sublingual space above the mylohyoid muscle that extended to the left submandibular and left parapharyngeal space displacing the left submandibular gland downward (Fig. 1). The preliminary report of needle aspiration at the submandibular area was an epidermoid inclusion cyst. The most likely diagnosis was a lateral sublingual epidermoid cyst. The patient was scheduled for surgical treatment. For a better cosmetic result, we chose an intraoral approach with the informed consent of the possibility of conversion to an extraoral approach if the intraoral approach was unsuccessful (Fig. 2). The patient underwent nasotracheal intubation. The left Wharton’s duct was cannulated with a lacrimal probe to prevent injury. An incision was made in the mucosa along the floor of the mouth. Because the sublingual gland obscured the view of the posterior Wharton’s duct, lingual nerve, and underlying cyst, the gland was excised to provide the necessary exposure. We then carefully separated the cyst from the mylohyoid and hyoglossus muscles by blunt dissection while preserving the lingual nerve and Wharton’s duct. The cyst was removed with an intact capsule (Fig. 3). The floor of the mouth was closed in layer with 3/0 Vicryl. During the postoperative period, the patient had only mild swelling at the floor of the mouth and intact tongue movement. She resumed a soft diet on the first postoperative day. The surgical specimen was a soft yellowish-tan cystic mass, measured 6.5 × 3.2 × 2.5 cm. with yellow keratin content. Histologic examination showed a benign stratified squamous epithelium-lined cyst with no evidence of adnexal structure consistent with the epidermoid cyst (Fig. 4). The patient recovered (Fig. 5) and remained disease-free after two years of follow-up.

## 3 Discussion

Dermoid cysts in the floor of the mouth are uncommon entities. New and Erich et al. found 7% of dermoid cysts in the head and neck area of which only 1.6 % occurred on the floor of the mouth [2]. The floor of the mouth dermoid cyst is the second most common for the head and neck region after the lateral eyebrow. Two pathogenesis theories of the dermoid cyst have been described. The first theory is acquired implantation in which there is a traumatic inclusion of epithelial cells within the underlying tissue or from occlusion of the sebaceous gland. But the lesions only present following the clear history of trauma or surgery. The second theory, which is more common, is the congenital inclusion cysts that are formed by the epithelial entrapment during midline fusion of the first and second pharyngeal arches during the third and fourth weeks of gestation. Meyer I et al. firstly classified floor of mouth dermoid cyst into three variants based on histopathology which was epidermoid, dermoid and teratoid variants [3]. In 2013, Gordon et al. proposed to use the terminology “congenital germline fusion cyst” instead of “dermoid cyst” to avoid confusion with the teratoma entity, which is the true solid neoplasm. They also recommended adding the specific Meyer’s variant behind this terminology to specify three the histopathologic subtypes which are 1) Epidermoid cyst: epithelial-lined cyst wall with no adnexal structure. 2) True dermoid cyst: epithelial-lined cyst with skin appendages (sebaceous and sweat gland and hair follicle) in cyst wall or compound cyst. 3) Teratoid cyst: epithelium lining with the derivative of all three germ layers (bone, muscle, gastrointestinal tissue) or complex cyst [4]. Of the three variants, epidermoid is the most common, followed by dermoid and teratoid, respectively. There is a bimodal age distribution that can first present during infancy and later during puberty when hormone change leads to increased sebum production, but they can occur at any age.

Anatomically, the floor of mouth dermoid cyst can be divided into three regions as sublingual, submental and lateral according to the anatomic relationship between cyst and floor of mouth muscle. The majority of the cysts (78 %) are in the midline, 16 % involve more than one space and only 6% are located in the submandibular space [5]. The signs and symptoms depend on the location of the cyst. Above the mylohyoid muscle, the clinical presentation is swelling at the floor of the mouth or posterior tongue displacement which can later cause swallowing, speaking and even airway problems. Beneath the mylohyoid muscle, the patient will present with a submental or submandibular mass depending on the laterality of the cyst. In our case presentation, although the location of the cyst is at lateral sublingual space above the mylohyoid muscle, it displaced the submandibular gland posteroinferior more than the above sublingual gland. This made the patient presented with submandibular swelling rather than the floor of mouth swelling. The important differential diagnosis includes plunging ranula, vascular or lymphatic malformation, chronic sialadenitis, and salivary gland neoplasm.

MRI imaging allows us to define the location of the cyst and its relationship to geniohyoid and mylohyoid muscle. This information is important for selecting the optimal surgical approach. Dermoid cysts are isointense or hypointense to muscle on T1-weighted image and hyperintense or heterogeneous on T2-weighted image.

Fine needle aspiration cytology is useful in differentiating the cyst as it did in our case. However, aspiration of the high viscosity content cyst may be difficult and inadequate for interpretation. A definite pathologic examination is essential for confirming the diagnosis.

Surgical excision is the treatment of choice. An aspiration to decompress the cyst before endotracheal intubation may be useful for a very large cyst obstructing the airway. The surgical approach can be an intraoral or extraoral approach depending on the size and location of the cyst. The intraoral approach is recommended for a small to a medium sublingual cyst which is less than 6 cm in size and above the mylohyoid muscle, whereas an extraoral approach is appropriate for large cysts over 6 cm located or transgress below the mylohyoid muscle [[5], [6], [7]]. Although the cyst in our case was large (6.5 cm) and extended behind mylohyoid to involve the submandibular and parapharyngeal space, we chose the intraoral approach to avoid cosmetic problems. Most literature reports the cases of midline sublingual dermoid cysts transorally excised by dissection through the midline raphe of the tongue and floor of the mouth [[8], [9], [10], [11]]. Gulati U et al. reported the combined intraoral and extraoral approach to remove large lateral sublingual epidermoid cyst [12]. They did the submandibular approach and devided the mylohyoid muscle to expose the inferior part of the cyst and additional approach from the floor of mouth to dissect the superomedial aspect. In our present case, the cyst was in lateral sublingual space with posterior extension to parapharyngeal space. We proposed to remove the sublingual gland for two reasons. Firstly, the posterior portion of the sublingual gland obscures the view of posterior part of Wharton’s duct, lingual nerve, and medial pterygoid muscle which are important structures needed to be preserved. After sublingual gland removal, there was more space to dissect the cyst from the surrounding muscle without disruption of the capsule. Secondly, the mucosal incision at lateral floor of mouth disrupts the duct opening of sublingual gland, if the gland is left, the mucocele or ranula will occur in the future. We report the first case of a large lateral sublingual epidermoid cyst with parapharyngeal extension removed by an intraoral approach. By excision of the sublingual gland first, an intraoral approach is possible for large lateral sublingual dermoid cysts that transgress mylohyoid muscle. The intraoral approach provided the patient with excellent cosmetic and functional outcomes. Recurrence is rare after complete excision but a 5% rate of malignant transformation of the dermoid and teratoid variant has been reported [[5], [6], [7]].

## 4 Conclusion

The epidermoid and dermoid cyst should be considered one of the differential diagnoses in the patient presented with a cystic mass in the floor of mouth or neck mass. Preoperative imaging is important for localizing the lesion and determining the surgical approach. Surgical excision by an intraoral approach is feasible and has an excellent cosmetic outcome.

## Funding

This research did not receive any specific grant from funding agencies in the public, commercial, or not-for-profit sectors.

## Ethical approval

This research has been approved by Research Ethics Committee 4, Faculty of Medicine, Chiang Mai University.

Study Code : ENT-2563-07145 / Research ID : ENT-2563-07145

## Consent

Patient/guardian consent was obtained for publication of this case report.

## Author contribution

Hanpon Klibngern: Conceptualization, Investigation, Resources, Manuscript writing and revision

Chanisa Pornchaisakuldee: Manuscript writing and revision, Manuscript submission

## Registration of research studies

1. Name of the registry: -

2. Unique identifying number or registration ID: -

3. Hyperlink to your specific registration (must be publicly accessible and will be checked): -

## Guarantor

Hanpon Klibngern

## Provenance and peer review

Not commissioned, externally peer-reviewed

## Declaration of Competing Interest

The authors declare that we have no conflict of interest.
